# Prevention of cell-surface attachment and reduction of penicillin-binding protein 2a (PBP2a) level in methicillin-resistant *Staphylococcus aureus* biofilms by *Acalypha wilkesiana*

**DOI:** 10.1186/s12906-015-0615-6

**Published:** 2015-03-25

**Authors:** Carolina Santiago, Kuan-Hon Lim, Hwei-San Loh, Kang Nee Ting

**Affiliations:** Faculty of Science, University of Nottingham Malaysia Campus, Jalan Broga, 43500 Semenyih, Selangor Malaysia

**Keywords:** MRSA, Biofilms, Cell attachment, PBP2a, *Acalypha wilkesiana*

## Abstract

**Background:**

Formation of biofilm is known to enhance the virulence of methicillin-resistance *Staphylococcus aureus* (MRSA), which is associated with persistent infections in hospital settings. The biofilm layer essentially forms a protective barrier encapsulating the bacterial colony and thus reduces the effectiveness of chemotherapeutics. We have isolated 9EA-FC-B bioactive fraction from *Acalypha wilkesiana* Müll. Arg. that reverses ampicillin resistant in MRSA through inhibition of the antibiotic resistant protein, penicillin-binding protein 2a (PBP2a). In this study, we aimed to investigate the effects of 9EA-FC-B on MRSA biofilm forming capacity.

**Methods:**

Inhibition of biofilm production and microtiter attachment assays were employed to study the anti-biofilm activity of 9EA-FC-B, while latex agglutination test was performed to investigate the effect on PBP2a in the biofilm matrix. We also attempted to characterise the chemical components of the fraction using high performance liquid chromatography (HPLC) and phytochemical analysis.

**Results:**

Fraction 9EA-FC-B and ampicillin exhibited similar inhibitory effect on MRSA’s biofilm production at their respective minimum inhibitory concentrations (81.56% vs 84.49%, respectively). However, the test fraction was more effective in suppressing cell surface attachment (90.85%) compared to ampicillin (37.8%). Interestingly, ampicillin enhanced the level PBP2a and in the contrary 9EA-FC-B attenuated the production of the resistant protein in the bioflim matrix. HPLC and phytochemical analysis revealed that 9EA-FC-B fraction is a complex mixture containing tannins, saponins, sterol/steroids, and glycosides.

**Conclusions:**

Bioactive fraction 9EA-FC-B inhibited the production of MRSA biofilm by preventing the initial cell-surface attachment and reducing the amount PBP2a in the matrix. PBP2a found in the biofilm matrix is believed to have a role in the development of virulence in MRSA.

## Background

The biofilm formation of MRSA has been attributed as a major factor for nosocomial infections [1] and treatments for these infections are further complicated by the presence of other virulent factors such as toxic production and host immune evasion ability [2]. A distinctive characteristic of biofilm or glycocalyx barrier is its recalcitrant to antimicrobial agents. The biofilm forms a physiological wall protecting bacterial cells from any fluctuations of the environment including any potential antibacterial agents [3,4]. Biofilm protected bacterial cells present a different mode of growth compared to planktonic cells, and the peculiarity of the mode of growth contributes to manifestation of antibiotic resistance. Due to this reason, treatment for biofilm-related infection becomes increasingly challenging, leading eventually to chronic device related infections which are often difficult to treat [3,5]. Most of the time, removal of the infected devices is the only clinical solution and the affected patients are succumbed to increased trauma as a result [6].

Biofilm formation in MRSA was previously reported to be mediated by the resistant protein, PBP2a, which is acquired and expressed in MRSA to overcome antimicrobial action of beta-lactam antibiotics [7]. It was hypothesized that PBP2a facilitates cell-cell interactions in the formation of MRSA biofilm [8]. Hence, development of anti-biofilm agents that interfere with steps involved in biofilm formation and disrupt PBP2a expression would be a sensible approach in developing a new adjunctive treatment for recalcitrant MRSA infections.

In recent reviews, plants have been identified as new sources of resistance-modifying agents based on their capacity of producing vast classes of antibacterial compounds [9-11]. Hence, a useful strategy in controlling MRSA infections is by identifying plant components that can inhibit biofilm production. *Acalypha wilkesiana* Müll. Arg. belonging to the Euphorbiaceae family has been traditionally utilized to treat bacterial and fungal infections, malaria, and gastrointestinal problems [12,13]. Previously, we found anti-MRSA and other biological activities from *A. wilkesiana* extracts and 9EA-FC-B [14,15]. This study aimed to investigate the effects of 9EA-FC-B on MRSA’s biofilm mechanism.

## Methods

### Plant material, isolation of bioactive fractions and determination of MIC

The plant material, *A. wilkesiana* was collected from Broga, Selangor, Malaysia and identified by Dr Christophe Wiart from the School of Pharmacy. Voucher samples was deposited in the herbarium of Faculty of Science, University of Nottingham Malaysia Campus and assigned as UNMC 9. The dried and ground plant materials (3.6 kg – *A. wilkesiana* whole plant) was subjected to sequential extraction using *n*-hexane (He), followed by ethyl acetate (EA) and finally 95% ethanol (EtOH). Extraction with each solvent was conducted by soaking the plant material in 10 L of the solvent (24 hours × 3 times) at room temperature.

The fraction 9EA-FC-B was isolated using a combination of vacuum liquid chromatography and preparative centrifugal thin layer chromatography (silica gel) using the following solvent system as the eluent: He with increasing amount of chloroform (CHCl_3_) and CHCl_3_ with increasing amount of methanol [15].

MICs of 9EA-FC-B and ampicillin against MRSA ATCC 43300 which was 3.00 mg/mL and 0.05 mg/mL respectively were determined previously using broth microdilution assay conducted at 35°C [15]. The strain used for this study (ATCC 43300) is a clinical strain that was found to be resistant to beta-lactam antibiotics [16-18] and was confirmed to be carrying the *SCCmec* type II chromosome with *mecA* gene regulator that is responsible for expression of PBP2a [3,19].

### Phytochemical and HPLC analysis

Phytochemical analysis of 9EA-FC-B was carried out according to the methods described previously [20]. An aliquot of 9EA-FC-B (40 μL of 10 mg/mL) was analyzed by C_18_-reversed phase HPLC using the following gradient solvent system: 2 min at 10% acetonitrile (ACN)/miliQ water (H_2_O); a linear gradient to 75% ACN/H_2_O over 12 min; isocratic at 75% for 10 min; a linear gradient to 100% ACN for 2 min; isocratic at 100% ACN for 4 min. HPLC was performed on a Varian 940-LC system using a reversed phase analytical column (Pursuit XRs C18, 4.6 × 150 mm, 5 μm) with photodiode array (PDA) detection at 254 nm.

### Bacterial strains and growth conditions

The bacterial strain used in this study was MRSA ATCC 43300. The strain was maintained on tryptic soy agar (TSA) (Hi-Media, India) supplemented with 2% NaCl (Merck, Germany). All the experiments were initiated using fresh overnight cultures grown in tryptic soy broth (TSB) (Hi-Media, India) containing 1% glucose (Merck, Germany).

### Preparation of test agents

Fraction 9EA-FC-B was dissolved in dimethyl sulfoxide (DMSO) (Sigma, USA) at stock concentration of 100 mg/mL. Further dilution was carried out using TSB and the final concentration of DMSO in the media did not exceed 1%. DMSO did not exert effect in the testing system as shown in our preceding work [15,21]. Ampicillin (Amresco, USA) was prepared at 10 mg/mL in sterile distilled water.

### Inhibition of biofilm production assay

This experiment was conducted according to Mataraci and Dosler [17] with a slight modification. A 96-well microtiter plate was prepared with 9EA-FC-B at the following concentrations: MIC (3.00 mg/mL), 1/2 × MIC (1.50 mg/mL), 1/4 × MIC (0.75 mg/mL), 1/8 × MIC (0.38 mg/mL), and 1/16 × MIC (0.19 mg/mL); and ampicillin at MIC (0.05 mg/mL). Aliquot of MRSA suspension was diluted with the media (TSB + 1% glucose) prior to the assay and was added to these wells. Final inoculums size was 1 × 10^5^ CFU/ml in total volume of 200 μl in each well. The plate was incubated for 24 h at 35°C. After incubation, the wells were washed with physiological buffered saline (PBS) solution and quantification of biofilm production was established by crystal violet staining method [22]. Briefly, the crystal violet staining method includes addition of 99% methanol (200 μl, Fisher Scientific Chemicals, USA) into each well for fixation of attached bacteria and followed by removal of the solvent and drying of the microtiter plate. Once dried, the wells of the microtiter plate were stained with 0.1% crystal violet (v/v in water) for 5 mins. The excess stain was discarded and plates were air dried. The stain was solubilised by adding 200 μl of 95% ethanol (Fisher Scientific Chemicals, USA). The optical density (OD) was read at 595 nm using a multimode plate reader (Varioskan Flash, Thermo Scientific, USA). Experiment was done in triplicates on three separate occasions.

### Microtiter attachment assay

The methods employed in this experiment have been described previously [23]. A 96-well microtiter plate was prepared with 9EA-FC-B and ampicillin at the same concentrations as in the inhibition of biofilm assay (see above). Aliquot of MRSA suspension was diluted with the media (TSB + 1% glucose) prior to the assay and was added to these wells. Final inoculums size was 1 × 10^7^ CFU/ml in total volume of 200 μl in each well. The plate was incubated for 1 h at 35°C. Following incubation, the wells were washed with PBS and the percentage of cell attachment was determined by the crystal staining method described above [22]. Experiment was done in triplicates on three separate occasions.

### PBP2a latex agglutination test on MRSA Biofilm

Prior to the latex agglutination test, MRSA was cultured in 50 mm diameter petri dishes in 10 mL of TSB + 1% glucose supplemented with 9EA-FC-B with concentrations ranging from 0.19-3.00 mg/mL and ampicillin at 0.05 mg/mL. The petri dishes were incubated for 24 h at 35°C. After incubation, the broth was carefully removed and 0.5 mL PBS was added to the petri dishes. Using a sterile 5 μl inoculating loop, the biofilm layer was scraped off just to fill the internal diameter (gives approximately 1.5 × 10^9^ CFU/ml). The obtained bacterial biofilm was processed according to the manufacturer’s instructions on the MRSA screening kit (Cat. no. DR900A Denka Seiken, Japan) in order to detect the presence of PBP2a. Semi-quantitative estimation of PBP2a production in biofilms was done based on the protocols described in Zhao et al. [24] in which the intensity of agglutination was observed and scored between + and + + +, where the control latex which showed no reactivity in the absence of PBP2a is considered as “-”.

### Statistical analysis

Results for biofilm attachment and inhibition assays were shown as means ± standard deviation of three independent experiments. A one-way analysis of variance with Bonferroni multiple comparison tests was used to compare difference between the control and treated groups. A *P* value of 0.001 was taken as statistically significant.

## Results

### Chemical characterization of fraction 9EA-FC-B

**Figure 1:**
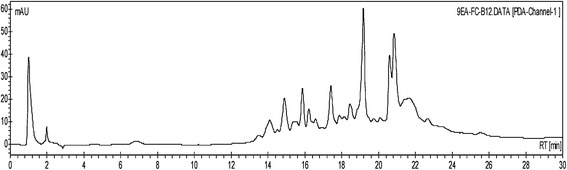
**HPLC chromatogram of 9EA-FC-B.** HPLC analysis of 9EA-FC-B (40 μL of 10 mg/mL, C_18_-reversed phase, 4.6 × 150 mm, 5 μm, detected at 254 nm) showing the presence of multiple severely overlapped peaks.

HPLC analysis revealed that 9EA-FC-B consisted of a complex mixture of compounds (Figure 1). These compounds were likely to be tannins, saponins, sterols/steroids, and glycosides based on the qualitative phytochemical analysis.

### Inhibition of MRSA biofilm production

**Figure 2:**
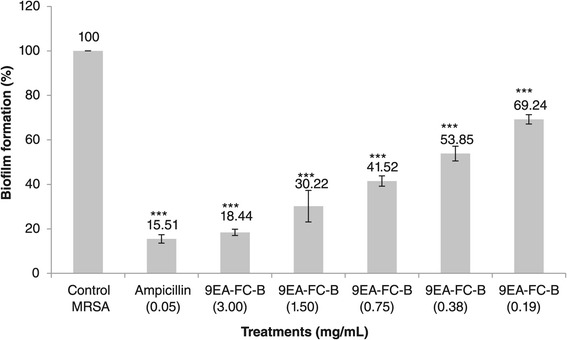
**MRSA biofilm formation (%) in 9EA-FC-B treatments.** Biofilm formation in microtiter plate wells containing 9EA-FC-B (mg/mL) at different concentrations. Three wells were used for each treatment. Experiment is representative of 3 independent tests, and error bars indicate the standard deviation. All difference between control and treated MRSA were statistically significant (*** -p < 0.001).

9EA-FC-B was tested at concentrations ranging from 3.00 mg/mL to 0.19 mg/mL and ampicillin at 0.05 mg/mL. The MICs against MRSA growth in planktonic state for 9EA-FC-B and ampicillin were 3.00 mg/mL and 0.05 mg/mL, respectively [15]. Figure 2 shows the percentage of MRSA biofilm formation in the different treatments. Generally, 9EA-FC-B exhibited appreciable activity against MRSA biofilm formation at MIC level with the biofilm formation reduced to just 18.44%. At concentrations lower than the MIC of 9EA-FC-B (i.e. 1.5 mg/mL and 0.75 mg/mL), the biofilm formation was reduced by more than 2-fold compared to control MRSA. On the other hand, ampicillin at MIC (0.05 mg/mL) reduced biofilm formation to 15.51%, comparable to that observed for 9EA-FC-B.

### Decreased cell-surface attachment

**Figure 3:**
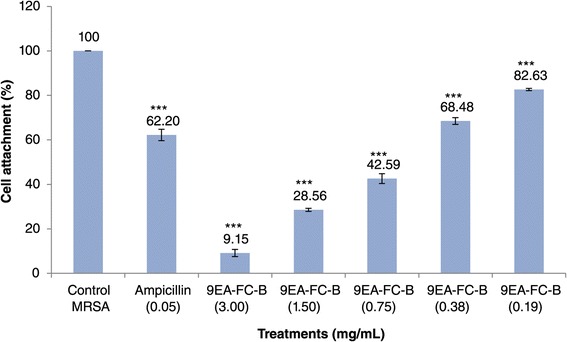
**Attachment of MRSA cells (%) to microtiter plate surface.** Attachment of MRSA cells in microtiter plate wells containing 9EA-FC-B (mg/mL) at different concentrations. Three wells were used for each treatment. Experiment is representative of 3 independent tests, and error bars indicate the standard deviation. All difference between control and treated MRSA were statistically significant (***-p < 0.001).

In order to elucidate the possible mechanism of 9EA-FC-B, cell-surface attachment was studied where MRSA cultures, treated either with ampicillin or 9EA-FC-B, were incubated for an hour. Cultures treated with 9EA-FC-B showed a concentration dependent reduction in cell-surface attachment. Notably, in the case of 9EA-FC-B at MIC, cell-surface attachment was markedly suppressed to 9.15%, but the same was not observed for ampicillin (62.20%) (Figure 3).

### Inhibition of PBP2a in MRSA biofilm

**Figure 4:**
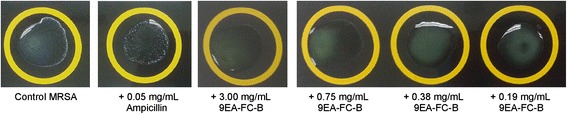
**Results of PBP2a latex agglutination test on MRSA biofilms.** A PBP2a latex agglutination test was performed and interpreted according to the manufacturer’s guidelines. Shown are MRSA biofilm samples from an untreated control MRSA and culture treated with MIC ampicilin (0.05 mg/mL), which had moderate and strong agglutination, respectively. The three disks from right are of biofilms samples from cultures treated with 9EA-FC-B at these concentrations; 0.19 mg/mL, 0.38 mg/mL and 0.75 mg/mL (as indicated) showing a very weak agglutination with turbid background. The middle disk is of biofilm sample from culture treated with MIC 9EA-FC-B (3.00 mg/mL) is showing no agglutination.

PBP2a latex agglutination test was conducted to measure semi-quantitatively the amount of the resistant protein, PBP2a found in the biofilm. A higher intensity of agglutination observed essentially corresponds to a higher level of PBP2a found in the biofilm. MRSA control showed a moderate intensity of agglutination while ampicillin treatment appeared to increase the amount of PBB2a in the biofilm. However, no PBP2a was detected for treatments with 9EA-FC-B at 3.00 mg/mL and 1.50 mg/mL (Table 1 and Figure 4).

**Table 1:** **Semi-quantitative estimation of PBP2a occurrence in biofilms isolated from different treatments**

**Treatments (mg/mL)**	**Control MRSA**	**Ampicillin 0.05**	**9EA- FC-B**				
			**3.00**	**1.50**	**0.75**	**0.38**	**0.19**
**Intensity of PBP2a Agglutination**	+ +	+ + +	-	-	+	+	+

Intensity of agglutination was observed and scored between + and + + +, where the control latex which showed no reactivity in the absence of PBP2a is considered as “-” (interpretation: + + + strong presence of agglutination, + + agglutination against turbid background, + slight agglutination against turbid background, − no agglutination).

## Discussion

In recent years, compound mixtures extracted from medicinal plants have demonstrated anti-biofilm activities against several virulent pathogens [25-27]. We have shown in this study that 9EA-FC-B exhibited potential anti-MRSA activity via inhibition of biofilm production. To further support the anti-MRSA effects of this plant we have reported on the effectiveness of this fraction to reverse ampicillin resistance by suppressing PBP2a expression [15]. Our earlier study has reported the non toxic effects of this plant against normal cell lines [14,28]. Therefore, suggesting that the components derived from this plant have specific toxicity against bacterial cells and cancer cells [28].

HPLC analysis revealed that fraction 9EA-FC-B is a complex mixture of plant metabolites, while phytochemical analysis showed a higher presence of tannins in the fraction compared to other phytochemicals (results not shown). This was corroborated by another group that previously reported the isolation of corilagin, geraniin and ellagitannin from the same plant and these tannin compounds demonstrated anti-staphylococcal activity. It was proposed that these tannins exert their antibacterial effects by causing cell wall damage which eventually results in cell lysis [29]. Besides, tannins were reported to be capable of binding to peptidoglycan and destroy the bacterial cell wall integrity [24]. The weaken cell integrity may hinder the initial phase of biofilm production that is the interaction between the bacterial cell wall and the surface [30-32]. It is therefore highly plausible that the occurrence of tannins in 9EA-FC-B was responsible for a strikingly reduced MRSA cell-surface attachment which was not observed in the ampicillin treated cultures. Since attachment of bacterial cells to the surface of its growth vessel influences the final mass of biofilm production, a reduced cell-surface attachment at the initial stage reduces the number of bacteria involved in biofilm development and production [23]. As such, prevention of cell-surface attachment by 9EA-FC-B was thought to have reduced MRSA biofilm formation. This was entirely consistent with the results from the inhibition of biofilm production assay that showed 9EA-FC-B inhibited biofilm formation.

Ampicillin on the other hand, was found to suppress MRSA biofilm formation comparable to the effect displayed by MIC of 9EA-FC-B. However, its inability to prevent cell-surface attachment within one hour of incubation in the microtiter attachment assay implied a possible delayed in its antibacterial action. Based on earlier reports, a delayed antimicrobial action contributes to prolonged and repeated exposure of MRSA to the antimicrobial agent which in return results in emergence of increased resistance [17,33]. This was further supported by our data from the PBP2a latex agglutination assay, which showed a higher level of PBP2a in the biofilms isolated from the MRSA cultures that were treated with ampicillin compared to the untreated control and those treated with 9EA-FC-B. Further support was provided by our recent finding based on Western blot experiments that showed treatment of MRSA cultures with ampicillin amplifies PBP2a expression in these bacterial cells [15].

Results of the semi-quantitative analysis of PBP2a latex agglutination test revealed that 9EA-FC-B reduced PBP2a level in MRSA biofilms. Earlier, we have demonstrated that 9EA-FC-B attenuated the level of PBP2a in MRSA based on Western blot experiments [15]. It was previously suggested that PBP2a mediates biofilm production in MRSA, while the altered cell wall structure of MRSA that expresses PBP2a promotes cell-cell interactions [8]. Although the mechanism by which PBP2a promotes MRSA biofilm production remains unclear it is known that cell-cell interaction is an important step in multilayer structure assembly in the development of biofilm [8,33]. Therefore, we propose that reduction of PBP2a level by 9EA-FC-B adversely affected cell-cell interaction and this, leads to a disruption in biofilm production.

## Conclusions

In summary, we have demonstrated that 9EA-FC-B affected the production of MRSA biofilm by preventing initial cell-surface attachment, disruption the cell-cell interaction and reducing the PBP2a concentration in the matrix. In addition to inhibiting the expression of PBP2a by 9EA-FC-B [15], the anti-biofilm activity of 9EA-FC-B represents another mechanism by which the fraction exerts its anti-MRSA action. Finally, the occurrence of tannins in 9EA-FC-B is postulated to be responsible for the observed anti-biofilm activity.
